# The effect of aberrant expression and genetic polymorphisms of Rad21 on cervical cancer biology

**DOI:** 10.1002/cam4.1592

**Published:** 2018-05-24

**Authors:** Li Xia, Minjie Wang, Hongying Li, Xiangjing Tang, Fei Chen, Jinquan Cui

**Affiliations:** ^1^ Department of Obstetrics and Gynecology The Second Affiliated Hospital of Zhengzhou University Zhengzhou China; ^2^ Department of Obstetrics and Gynecology People’s Hospital of Linying Luohe China; ^3^ Department of Obstetrics and Gynecology Pingdingshan First People’s Hospital of Henan Province Pingdingshan China; ^4^ Department of Gynaecology and Obstetrics Peking Union Medical College Hospital Chinese Academy of Medical Sciences and Peking Union Medical College Beijing China

**Keywords:** cervical carcinoma, Rad21, single‐nucleotide polymorphism, The Cancer Genome Atlas, XPO1

## Abstract

The therapeutic challenge of advanced, recurrent, and refractory cervical cancer (CC) needs to develop new molecularly targeted drugs. Rad21 is an important regulatory gene that maintains the correct dissociation of sister chromatids during cell mitosis. The aim of this study was to investigate the effect of Rad21 on CC. Rad21 expression in CC and cervical intraepithelial neoplasia III was significantly increased. Women with the rs2289937 C genotype (CC+CT) of rs4570 and rs4579555 genotypes and haplotype 1 (TTTCAGGCGC) were significantly associated with CC risk, while women with low frequencies of haplotype 6 (TTTTAGGCGC) also increased the risk of CC.Rad21‐specific shRNA decreased cancerous cell proliferation, migration, and invasion and increased the proportion of cells in G2/M phase as well as sensitivity to radiation. The Rad21 influenced the expression of XPO1, CyclinB1, CDK1, P21, P27, and P53 through up‐and downregulating the Rad21 expression. The TCGA database of CC also showed that Rad21 expression was associated with poor disease survival and XPO1 expression. Moreover, the KEGG pathway indicated that Rad21 is broadly involved in the cell cycle and RNA transportation via XPO1. This suggests that Rad21 involves the development of cervical cancer possibly by participating in the regulation of cell cycle and the nuclear output of the tumor suppressor gene via XPO1.

## 1 INTRODUCTION

Cervical cancer (CC) is one of the most common cancers of the female reproductive system worldwide. Recurrent and metastatic cancers are difficult to treat. Chemoradiotherapy is a main course of treatment for those patients; however, the effect is limited due to the repair of chemoradiotherapy‐induced DNA damage in tumor tissues. It has been confirmed in preclinical and clinical trials that inhibition of the DNA damage response increases the chemo (radio) sensitivity of CC cells and improves the efficacy of cancer therapy.^1^ Moreover, recent progress in molecular genetics has demonstrated that gene polymorphisms (eg, single‐nucleotide polymorphisms, SNPs) are associated with multiple biological processes, and specific combinations of SNPs are involved in the carcinogenic process. Persistent infection of high‐risk HPV has been considered as a main cause of CC. Genetic factors such as gene SNPs may participate in the malignant transformation of HPV‐infected cells to CC onset.^2^ SNP changes also have the potential to predict clinical responses to chemoradiotherapy and the disease progression of CC.^3^

The ring‐shaped cohesin complex plays an important role in many aspects of chromosome biology, including sister chromatid cohesion, DNA repair, and transcriptional regulation. Rad21 protein, one of the 4 major subunits of the cohesin complex, is widely involved in the regulation of physiological function in the human body, and its expression profile is heterogeneous in different malignant tumors.^4^ Recently, Rad21 was found to be associated with the development and prognosis of malignant tumors. Xu et al^5^ showed that the expression of Rad21 in breast cancer is related to the early recurrence of breast cancer. Additionally, high Rad21 expression in patients with high‐grade luminal, basal, and human epidermal growth factor receptor 2 (HER2)‐positive breast cancers shortens the overall survival of patients and decreases chemosensitivity. In colon cancer, Deb et al^6^ conducted a retrospective study of 652 patients with colon cancer and found that Rad21 expression is related to disease progression, showing a positive correlation with chemotherapy in tumors harboring KRAS mutations and resistance. Moreover, Xu et al^7^ showed that activation of the Wnt pathway promotes Rad21 gene expression, and elevated Rad21 expression tracks with reactivation of LINE‐1 expression in human sporadic colorectal cancer. However, the role of Rad21 in CC has not yet been reported.

Accordingly, in this study, we detected Rad21 expression in Siha and HeLa cells and CC tissues by immunohistochemistry and assessed the role of Rad21 in CC through the analysis of genetic polymorphisms as well as the evaluation of the biological effects of Rad21 knockdown in CC cells. The TCGA RNA sequencing database of CC showed that Rad21 was associated with poor disease survive and XPO1 expression. Our findings provide important insights into the functions of Rad21 as a potential therapeutic or prognostic target in CC.

## 2 MATERIALS AND METHODS

### 2.1 Patients and samples

One hundred and ten archived wax blocks were collected for immunohistochemical analysis from the Second Affiliated Hospital of Zhengzhou University from August 5, 2009 to May 1, 2010. The sample tissues were categorized as follows: 15 normal cervix, 8 cervical intraepithelial neoplasia (CIN) Is, 10 CINIIs, 12 CINIIIs, and 65 CCs. To analyze the gene susceptibility of CC, 120 patients with CC diagnosed at the Second Affiliated Hospital of Zhengzhou University were enrolled as the case group, and 120 patients without cervical malignant tumors or precancerous lesions were enrolled as the control group from March 2010 to October 2011; all patients were of Han ethnicity. Two milliliters of peripheral blood was collected into sodium citrate‐containing tubes and stored in a refrigerator for nucleotide polymorphism analysis. In both study populations, no patients underwent surgery, radiotherapy, or chemotherapy prior to sample collection; clinical data were accurate and complete. The experimental protocol was established according to the ethical guidelines of the Declaration of Helsinki and was approved by the Human Ethics Committee of The Second Affiliated Hospital of Zhengzhou University.

### 2.2 CC data from The Cancer Genome Atlas (TCGA) database

The clinical and pathological data of patients with CC retrieved from TCGA database (https://tcga-data.nci.nih.gov/docs/publications/) included 306 cancer samples and 3 normal samples. R3.3.2 software (https://www.r-project.org/) was used to download and analyze the patients’ clinical data and mRNA expression in cancer tissues.

### 2.3 Cell culture

HeLa and Siha human CC cells were obtained from the Institute of Medical Experimental Animals of the Chinese Academy of Medical Sciences (Shanghai, China). Primary normal epithelial cells (NC) were cultured from fresh human cervical tissues donated by 3 patients (aged 56, 58, and 46 years, respectively) in Department of gynecology of the Second Affiliated Hospital of Zhengzhou University in February 2018, after hysterectomy for benign uterine diseases. All the patients were tested negative for HPV and Liquid‐based cytology (LBC) before surgery, and the postoperative pathological examination showed no abnormal condition. The culture medium was a mixture of defined keratinocyte serum‐free medium (Gibco, America) and 5% FBS (Gibco, America) with the provided supplements. The cells were subcultured at a split ratio of 1:3 when they reached 70%‐80% confluency. Population doublings (PD) were calculated after the first subculture from primary cells (PD0). At PD3, the Western blot test was used to detect Rad21 protein expression.

### 2.4 Immunohistochemistry

Rad21 expression was analyzed in paraffin sections using rabbit anti‐Rad21 antibodies (1:200 dilution, Abcam) and an immunohistochemistry assay kit (Origene). Positive immunostaining of Rad21 appeared as a brown or tan color in the nucleus or cytoplasm of cancer cells. According to the proportion of positive cells and staining intensity, the expression level was scored. Expression was considered negative when the total score was 0‐2 and positive when the total score was more than 2.

### 2.5 Tag single‐nucleotide polymorphism (SNP) detection in the Rad21 gene and screening of SNP loci

DNA from blood samples was extracted according to the manufacturer’s instructions (Thermo). After PCR amplification of DNA samples, PCR production was transferred to a Massarray Spectrochip system (SEQUENOM). Ten tag SNPs of the Rad21 gene were detected by matrix‐assisted laser desorption ionization time‐of‐flight mass spectrometry (SEQUENOM). The SNPs included rs4570, rs16888927, rs3816342, rs2289937, rs2921785, rs16888997, rs16889040, rs4579555, rs6987652, and rs10107209.

### 2.6 Packaging and screening of interfering lentiviral expression systems

The lentivirus expression system and packaging kit included 4 specific shRNAs and one scrambled negative control plasma sample. The template sequences of 4 specific shRNAs targeting Rad21 were as follows:

Rad21shRNA(TL309968A), AACAGTTCAGAACAGATGTGTGCAATATT; Rad21shRNA(TL309968B), ACCTGCTCAGCCTTTGTGGAATAACAGAC; Rad21shRNA(TL309968C), GCTATTGAGCTGACACAGGAAGAACCGTA; Rad21shRNA(TL309968D), GGAAGCAGCTTATAATGCCATTACTTTAC. The experiment was performed strictly in accordance with the product instructions (Origene). Four shRNAs and one scrambled control shRNA were transfected into 293T cells using Opti‐MEM (Life Technologies). Then, viral supernatant from the culture was harvested and filtered for further transduction applications. The lentiviruses‐infected HeLa and Siha cells were seeded in 6‐well plates overnight. The cells were collected, spun, and suspended in the complete culture solution with 2.0 μg/mL puromycin. The infected cells were continuously cultured in a 2.0 μg/ml puromycin environment and stably expressed the lentiviruses.

### 2.7 Real‐time quantitative reverse transcription (RT)‐PCR

For RT‐PCR, total RNA was extracted from the cancer cells. After cDNA synthesis, PCR amplification was carried out using the following amplification conditions: 50°C for 2 minutes, 95°C for 10 minutes, and 47 cycles of 95°C for 15 seconds, 60°C for 30 seconds, and 72°C for 31 seconds. Sequence‐specific primers for Rad21 and β‐actin were designed and synthetized by Shanghai SANGON Biological Engineering Ltd. The primer sequences were Rad21, forward, AAACAAGCTGCCGCAAAGTT and reverse, TCCAGGTGTTGCGATGAT; β‐actin, forward, AGAAGGCTGGGGCTCATTTG and reverse, AGGGGCCATCCACAGTCTTC. β‐actin was used as the internal control, and the relative expression was calculated using the 2^−ΔΔCT^ method. Samples were assessed in triplicate, and the experiment was repeated 3 times.

### 2.8 Western blotting

Cell lysates were prepared using RIPA buffer containing the phosphatase inhibitor phenylmethylsulfonyl fluoride (100:1). Sodium dodecyl sulfate polyacrylamide gel electrophoresis was conducted to separate proteins on 8% gels, and proteins were then transferred to nitrocellulose membranes and blocked with 5% skim milk. Membranes were incubated with primary antibodies (Rad21, XPO1, CDK1, CyclinB1, P21, P27, P53 diluted in 1% skim milk) overnight at 4°C. Antibody incubation concentration: Rad21 and β‐actin: (1:3000, Abcam),XPO1: (1:10 000, Abcam),CDK1, CyclinB1, P21 and P27:(1:1000, Abcam), P53:(1:5000, Abcam).After washing, the membranes were incubated with secondary antibodies (1:5000) at 25°C for 1.5 hour, exposed and imaged with a FluorChem (FC3) chemiluminescence science imager. The gray protein bands were analyzed with ImageJ software, and β‐actin was used as the internal control for calculation of the relative expression level of Rad21 protein. The experiment was repeated 3 times.

### 2.9 Analysis of cell proliferation by CCK‐8 assays

During the logarithmic growth phase, CC cells were collected by trypsinization, and the cell concentration was adjusted. 2000 cells in 100 μL medium for each well were seeded in 96‐well plates, with 3 replicates per treatment. After 0, 24, 48, 72, or 96 hours, the culture medium was removed and replaced with 100 mL of Cell Counting Kit‐8 (CCK‐8) working solution (Shanghai Beyotime Biotechnology Co., Ltd.) diluted with fresh culture medium without serum (1:9). The cells were cultured for an additional 2 hours. The absorbance at 450 nm was measured, and cell growth curves were drawn based on the average of each sample.

### 2.10 Detection of cell apoptosis and cell cycle distribution

The cells were seeded into 6‐well plates and cultured for 48 hours. Flow cytometry was used to detect cell apoptosis and cell cycle in different groups according to the manufacturer’s instructions (BD). The experiment was repeated 3 times.

### 2.11 Wound healing assays

Straight lines 0.5 cm apart from each other were marked on the underside of 35 mm culture dishes. Logarithmic‐phase cells were seeded into the dishes. Nicks were made 0.5 cm apart on the inside bottom of the dish along with the labeled lines with a 200 μL sterile pipette tip after cells were close to confluent. The cultures were then washed with sterile PBS 3 times and incubated for 24 hours in 2% FBS medium. The scratch width was photographed under phase contrast on an inverted microscope with a digital camera (100× magnification). The experiment was repeated 3 times.

### 2.12 Cell invasion assays

Matrigel (Corning) was melted and mixed with cold 1640 medium at a ratio of 1:4. Transwell chambers were coated with the Matrigel solution. Cells were collected by trypsinization and adjusted to a concentration of 1×10^6^ cells/mL. 100 μL of the cell suspension was added to the upper chamber, and 600 μL of medium containing 10% fetal bovine serum was added into the lower chamber. After 24‐hours incubation, cells were fixed with 4% paraformaldehyde and stained with 0.5% crystal violet. The chambers were visualized under an inverted microscope, and 5 visual fields were randomly imaged at high magnification. The cells dyed purple were counted. Three wells were analyzed for each experiment, and the experiment was repeated 3 times.

### 2.13 Detection of radiosensitivity in clonogenic assays

The cells were diluted to 200 cells/mL, and 2 mL of the cell suspension was inoculated into 6‐well plates overnight in an incubator. Cells were then irradiated with 0, 2, 4, or 6 Gy. When cell colonies formed, they were fixed with methanol and dyed with 0.1% crystal violet. The number of clones (more than 50 cells/colony) was counted. The relative survival rate of cells was calculated according to the following formula: clone formation rate = number of clones/number of inoculated cells × 100%.

### 2.14 Rad21 gene transfection

Experimental cell is divided into 3 groups: pcDNA3.1‐Rad21 group (transfected with expressed Rad21 pcDNA3.1 plasmid), pcDNA3.1 group (transfected with pcDNA3.1 plasmid) and control group (normal HeLa cells). All plasmids were provided by Shanghai GenePharma Company and transfected into HeLa cells by Lipfectamin 2000 (Invitrogen, United States) according to the manufacturer^,^s instruction. Briefly, HeLa cells were suspended to a final concentration of 1 × 10^6^/mL and planted into a 6‐well plate. When the cells grew at 80% to 90% confluence, the medium was replaced with serum‐free medium. The pcDNA3.1‐Rad21/RPMI 1640 (4 μg:250 μL) and Lipfectamin 2000/RPMI 1640 (8 μL:250 μL) were mixed at room temperature for 15 minutes. Then added the complex into the cells, and the medium was replaced 24 hours later with antibiotic‐free medium containing 10% FBS. After 48 hours transfection, Western Blot was performed to detect Rad21expression levels.

### 2.15 Statistical analysis

Haploview software and SPSS17.0 statistical software were used for the statistical analysis. The measurement data are shown as the mean ± standard deviation. Differences in proportions were evaluated by χ^2^ tests. Univariate analysis of variance was used to compare data between groups. Results with *P*‐values of less than .05 were considered statistically significant.

## 3 RESULTS

### 3.1 Patient clinic pathological features

In total, 110 blocks of normal tissues or CIN/cancer tissues were used for the immunohistochemical analysis. Patients ranged in age from 31 to 79 years old, with an average age of 51 years old. According to the clinical staging criteria revised by the International Federation of Obstetrics and Gynecology (2009), of the 65 cases of squamous cell carcinoma, there were 10 cases of stage Ia, 16 cases of stage Ib, 22 cases of stage IIa, and 17 cases of stage IIb.

For patients enrolled in the genetic polymorphisms study, the average ages in the case group and control group were 48.9 years old (range: 30‐71 years) and 47.5 years old (range: 28‐68 years), respectively; there was no significant difference in age between the 2 groups. The case group included 106 squamous carcinoma patients and 14 adenocarcinoma patients.

### 3.2 Expression of Rad21 protein in different types of cervical tissues

**Figure 1:**
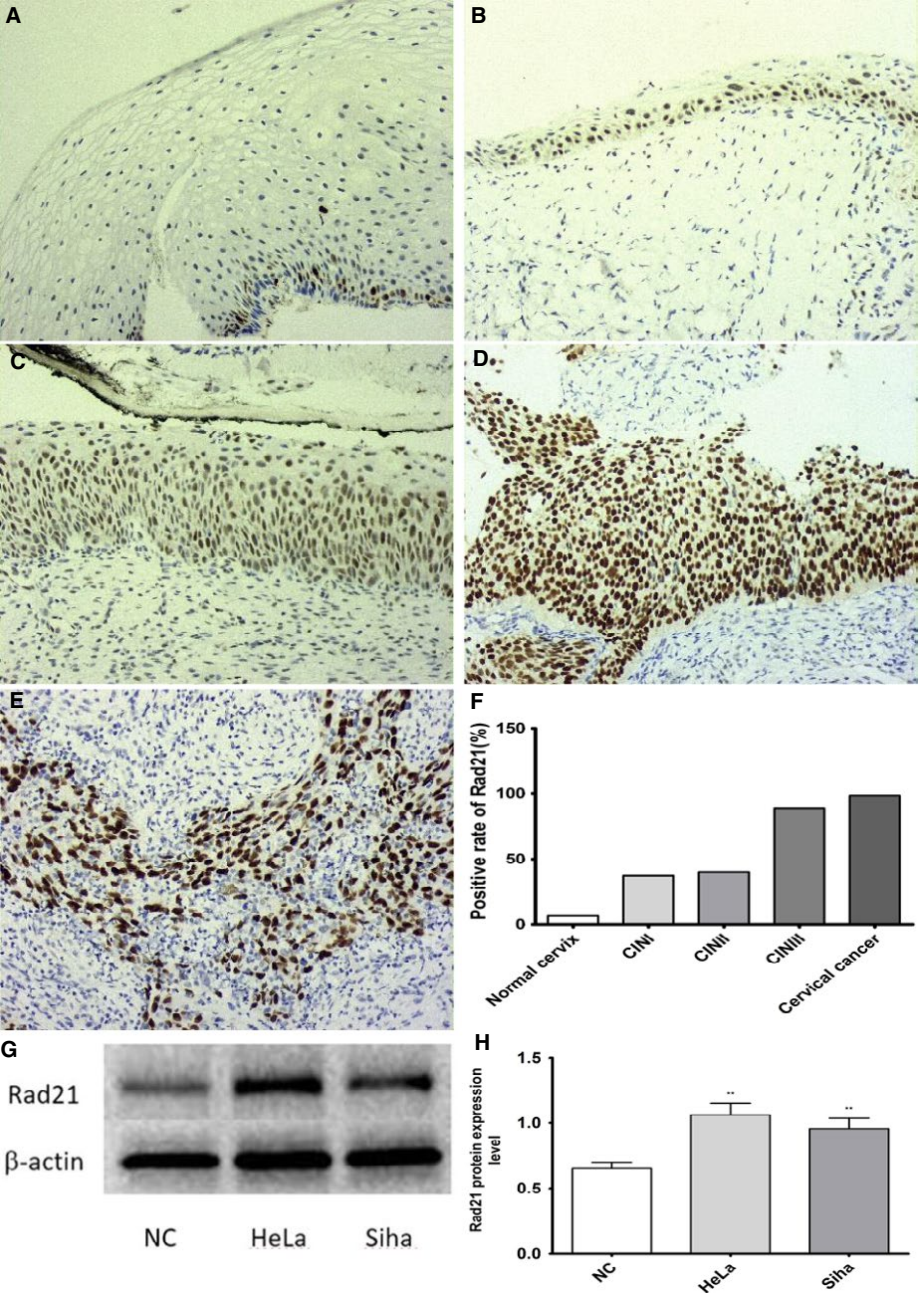
The expression of Rad21 protein in different cervical tissues and cell lines. Immunohistochemistry and Western blot were used to detect the expression of Rad21. A, normal cervix (200×) B, CINI (200×) C, CINII (200×) D, CIN III (200×) E, cervical squamous cell carcinoma F, the positive rate of Rad21 in different groups was significantly different (*P *< .001) G, the Rad21 protein expression of HeLa, Siha and primary normal epithelial cells (NC) H, with a significant difference between the groups (*P *< .01)

The positive Rad21 immunostain was primarily located in the nucleus of cervical epithelial cells. Small amounts of pale yellow granules were observed in scattered epithelial cells of the normal cervix (Figure 1A). CIN tissues showed medium to strong staining (Figure 1B‐D). CC tissues (Figure 1E) displayed strong staining with yellow or brownish yellow granules. The positive expression rates of Rad21 in normal cervix, CINI, CINII, CINIII, and CC were 6.60%, 37.50%, 40.40%, 89.20%, and 98.46%, respectively, with a significant static difference between the groups (*P *< .001, Figure 1F).The Rad21 protein expression of HeLa and Siha was higher than that of primary normal epithelial cells (NC), with a significant difference between the groups (*P *< .01, Figure 1G,H).

### 3.3 Association between Rad21 gene polymorphisms and CC susceptibility

The observed and expected values of all SNP alleles tested in this study are well fitted and conformed to the Hardy‐Weinberg equilibrium law (*P *> .001). The frequency of rs2289937C allelotypes in the women with CC was significantly higher than in the normal women (93.2% vs 86.7%, χ^2^ = 5.637 *P *= .0176, OR 2.115, 95% CI 1.127‐3.969). The distributions of other SNPs, ie, rs4570, rs16888927, rs3816342, rs2921785, rs16888997, rs16889040, rs4579555, rs6987652, and rs10107209, did not differ significantly between the case and control groups. The risk genotypes (CC + CT) of rs4570 and rs4579555 in the case group were significantly higher than the control group (OR 1.810, 95% CI 1.076‐3.043; OR 1.810, 95% CI 1.076‐3.043, respectively). Based on gene linkage disequilibrium of Rad21, 6 haplotypes were deduced (Table 1). The frequencies of haplotype 1 (TTTCAGGCGC) in the women with CC was significantly higher than the normal women, haplotype 6 (TTTTAGGCGC) in the women with CC was significantly lower than the normal women (all *P *< .05).

**Table 1:** Distribution of polymorphic haplotypes of the *Rad21* gene in the case and control groups

Haplotype	Content	Groups	Cases	Haplotype frequency	χ^2^	*P*	OR	95% CI
H1	TTTCAGGCGC	Case	118	0.347	4.479	.**0343**	1.529	1.031‐2.267
		Control	120	0.258				
H2	TCTCAAATAT	Case	118	0.212	0.102	.7489	1.075	0.689‐1.677
		Control	120	0.200				
H3	CTTCAGGTGC	Case	118	0.203	0.307	.5795	1.137	0.721‐1.793
		Control	120	0.183				
H4	TTCCAGGTGC	Case	118	0.161	2.356	.1248	0.694	0.437‐1.103
		Control	120	0.216				
H5	TTTTTGGTGC	Case	118	0.042	1.360	.2435	0.619	0.275‐1.394
		Control	120	0.067				
H6	TTTTAGGCGC	Case	118	0.025	4.591	.**0321**	0.365	0.140‐0.950
		Control	120	0.067				

Bold values: The frequencies of H1 in the women with CC was significantly higher than the normal women, H6 in the women with CC was significantly lower than the normal women (all *P* < .05).

### 3.4 Rad21 shRNA lentivirus infection downregulated Rad21 expression in HeLa and Siha cells

**Figure 2:**
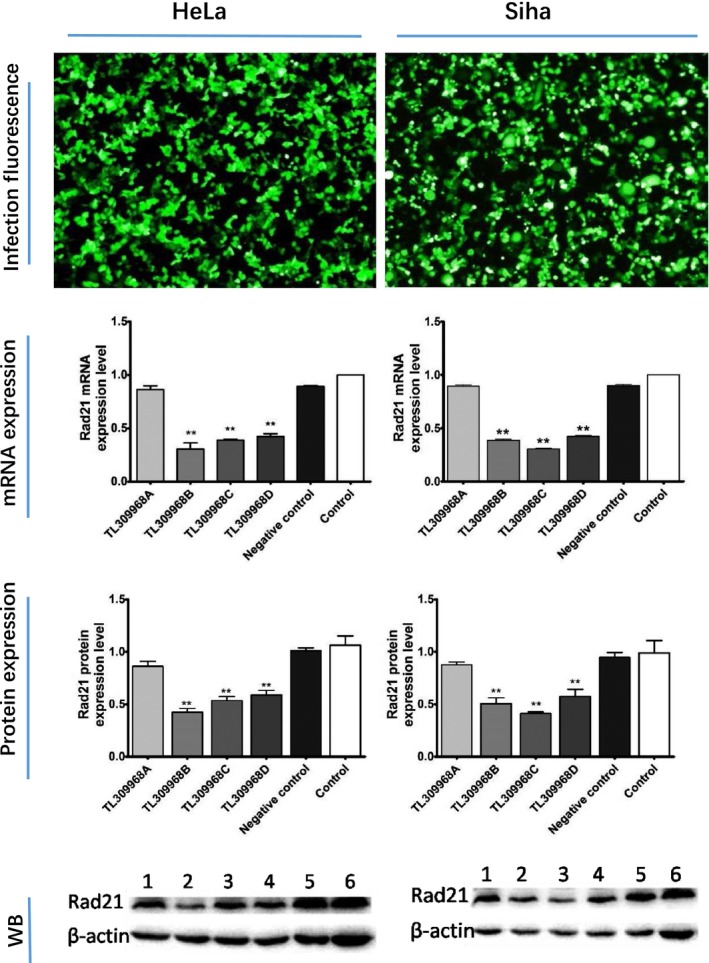
The knockdown effect of Rad21 shRNA on HeLa and Siha cells. Line 1, 2, 3, 4, 5, 6 represent Rad21 shRNA of TL309968A, TL309968B, TL309968C, TL309968D, scrambled negative control and control, respectively. **P < .01 vs the control group

Under a fluorescence microscope, large green fluorescent particles were found in the stably infected cells, with an infection rate of 70% (Figure 2). With the exception of TL309968A, the lentiviruses showed significant interference effects after 72 hours culture. The knockdown effect of TL309968B shRNA on the expression of Rad21 was the most effective in HeLa cells, whereas TL309968C shRNA was the most effective in Siha cells. The best knockdown efficiency of both shRNAs on mRNA was 69.52% and 69.70%, and the efficiency on protein expression was 60.00% and 58.33%, respectively.

### 3.5 Effect of Rad21 shRNA lentivirus on cell proliferation, apoptosis, and the cell cycle

The results of the CCK‐8 assays showed that the proliferation ability of HeLa and Siha cells was significantly inhibited by Rad21 knockdown in a time‐dependent manner (Figure S1). The best inhibition rates against the HeLa and Siha cells were 40.69% and 40.28%, respectively, after 4‐day culture. The proportions of apoptotic cells and cell cycles were detected by flow cytometry. After Rad21 knockdown for 48 hours, the apoptosis rates in HeLa and Siha cells were 13% ± 0.76% and 17.40% ± 0.57%, respectively; these values were significantly higher than those in the control groups (HeLa, 7.10% ± 0.45%; Siha, 9.45% ± 0.45%; all *P *< .01). The distributions of the G_2_/M phase of HeLa cells (13.66% ± 0.95%) and Siha cells (12.56% ± 0.86%) in the knockdown groups were significantly higher than those in the negative control groups (HeLa, 7.97% ± 1.56%; Siha, 7.62% ± 0.81%; all *P *< .01). Thus, these data demonstrated that cells were arrested in the G_2_/M phase (Figure S2).

### 3.6 Effects of Rad21 shRNA lentivirus on cell migration, invasion, and radiosensitivity

**Figure 3:**
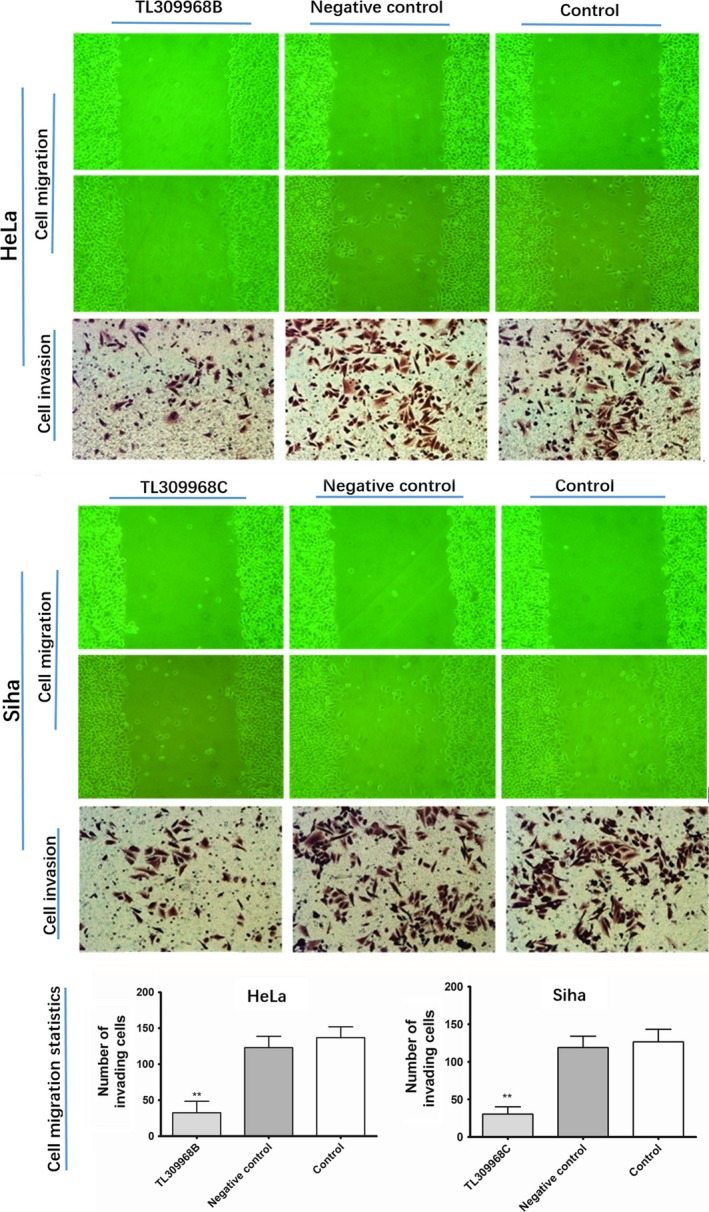
The migration and invasion ability of HeLa and Siha cells following knockdown of Rad21. After 24 h of culture, the migration ability of HeLa cells stably expressing TL309968B of Rad21 shRNA and Siha cells stably expressing TL309968C of Rad21 shRNA as viewed under an inverted microscope at 200× magnification was reduced. The invasion ability was detected using crystal violet staining (400×). **P < .01 versus the scrambled negative control

**Figure 4:**
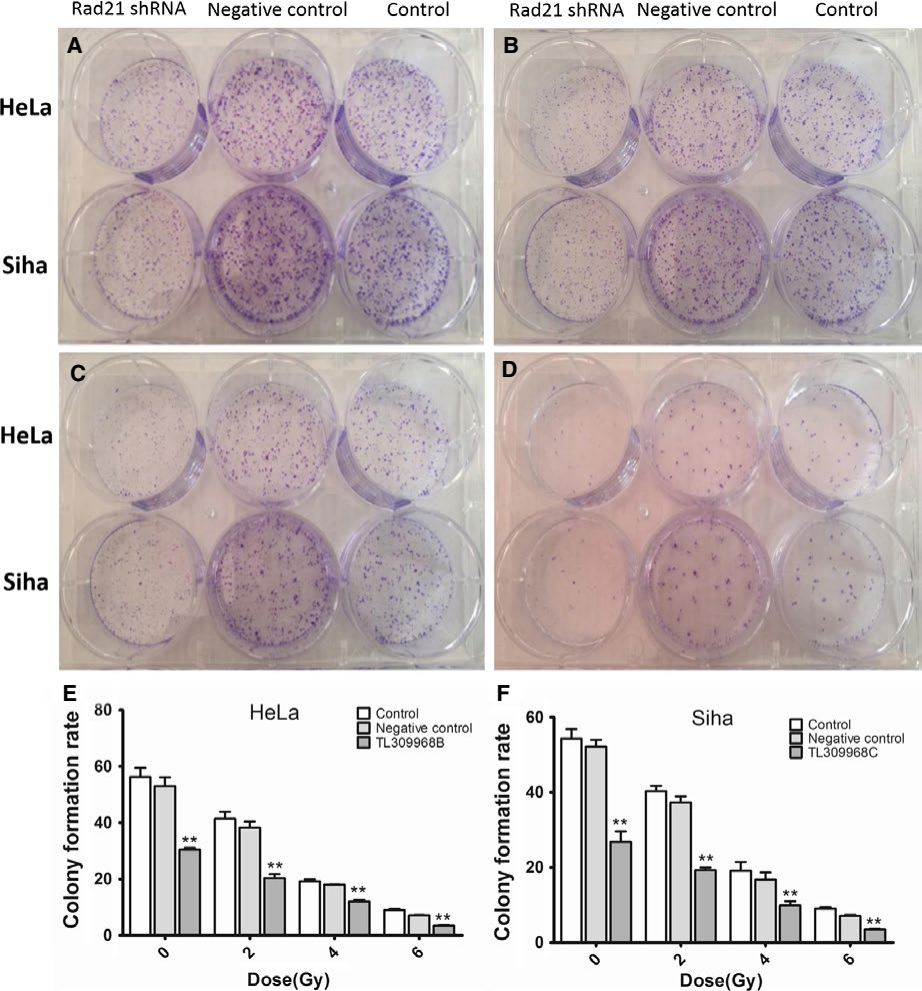
The effect of Rad21 shRNA on irradiation sensitivity. With the increase in the radiation dose, colony formation rates decreased after 48 h of culture. HeLa cells stably expressing TL309968B and Siha cells stably expressing TL309968C significantly increased the sensitivity to irradiation, and decreased colony formation rates (E, F). A, 0 Gy B, 2 Gy C, 4 Gy D, 6 Gy. **P < .01 vs the control, scrambled negative control, or 0 Gy

As shown in the wound healing assay, the migration ability of HeLa and Siha cells was blocked following Rad21 knockdown for 24 hours (Figure 3). Similarly, the invasion assay showed that Rad21 knockdown for 48 hours significantly blocked cell invasion in both HeLa and Siha cells (all *P *< .01; Figure 3). The sensitivities of HeLa and Siha cells to radiation were detected by clonogenic assays. As the dose of radiation increased, clonal formation in HeLa and Siha cells decreased gradually compared to that of the control group. The irradiation sensitivity of HeLa cells stably expressing TL309968B and Siha cells stably expressing TL309968C significantly increased (all *P *< .01; Figure 4).

### 3.7 Analysis of Rad21 expression in CC using TCGA database

**Figure 5:**
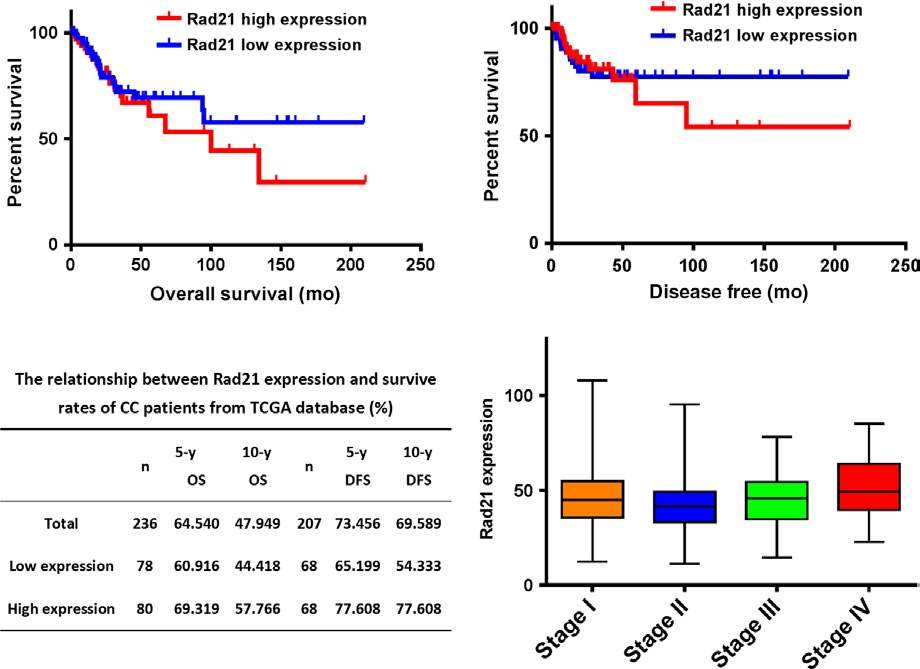
The association of Rad21 expression with the patient survival and disease stage of squamous cervical carcinomas. High expression of Rad21 was related to poor survival and advanced stages but was not significantly different

For the survival analysis, all patients were evenly divided according to Rad21 gene expression levels into low, middle and high groups. After excluding 4 cases with Rad21 mutations and patients with a lack of follow‐up data or without valid data, 236 patients with squamous cell carcinoma entered the overall survive (OS) analysis, and 207 patients for disease‐free survive (DFS). A Kaplan‐Meier survival analysis was performed to calculate, respectively, the 5‐ and 10‐year survival rates in the total, high‐expression, and low‐expression groups (Figure 5). The overall and survival time in the high‐expression group was lower than that in the low‐expression group (Figure 5). As shown in Figure 5, as the stage increased, Rad21 expression gradually increased, but without significant difference.

With a Spearman correlation coefficient value of ±0.4, 181 genes were positively correlated with Rad21 expression, and 35 genes were negatively correlated. Gene Ontology (GO) and Kyoto Encyclopedia of Genes and Genomes (KEGG) pathway analysis were performed between Rad21 and its coexpressed genes. GO analysis (Table 2) showed that Rad21 is located at chromosomal centromere sites, nuclear chromatin and the cohesin complex, with the capability of binding to chromatin and DNA‐dependent ATP enzyme activity. These results implied that Rad21 plays an important role in DNA recombination, double‐strand break repair, polymerization and the separation of mitotic sister chromatids. The protein interaction network of Rad21 and its coexpressed genes was mapped (Figure S3). KEGG pathway analysis (Figure S4) showed that Rad21 was associated with the cell cycle and RNA transport.^8^, ^9^ CRM1/chromosome region stabilizing protein (XPO1, export protein 1) is involved in RNA transport under the action of RNA polymerase, whose function is highly related to Rad21 protein expression (Figures S3 and S4).

**Table 2:** Gene Ontology enrichment analysis of the genes coexpressed with Rad21

ID	Description	Term type	*P*‐value	*P*‐adjust	DEG item	BG item
GO:0098687	Chromosomal region	CC	4.90E−11	6.85E−09	22	341
GO:0000775	Chromosome, centromeric region	CC	2.83E−09	2.64E−07	15	184
GO:0000793	Condensed chromosome	CC	7.59E−08	4.25E−06	14	202
GO:0044454	Nuclear chromosome part	CC	3.81E−06	.000106704	19	496
GO:0000785	Chromatin	CC	.000193746	.002855203	15	450
GO:0000794	Condensed nuclear chromosome	CC	.000459619	.005849693	6	87
GO:0008278	Cohesin complex	CC	.005399784	.047248114	2	10
GO:0016887	ATPase activity	MF	2.16E−06	.000437105	18	423
GO:0008094	DNA‐dependent ATPase activity	MF	2.70E−06	.000437105	8	76
GO:0003777	Microtubule motor activity	MF	.000268085	.022289642	6	77
GO:0003678	DNA helicase activity	MF	.000275181	.022289642	5	50
GO:0015631	Tubulin binding	MF	.000548405	.035536676	11	288
GO:0008017	Microtubule binding	MF	.000889798	.039581254	9	214
GO:0008574	ATP‐dependent microtubule motor activity, plus‐end‐directed	MF	.000924079	.039581254	3	17
GO:0042623	ATPase activity, coupled	MF	.001084829	.039581254	11	313
GO:1990939	ATP‐dependent microtubule motor activity	MF	.001099479	.039581254	3	18
GO:0003682	Chromatin binding	MF	.001281803	.041530427	14	474
GO:0007067	Mitotic nuclear division	BP	3.42E−18	7.57E−15	33	429
GO:0007059	Chromosome segregation	BP	4.00E−15	2.21E−12	26	318
GO:0098813	Nuclear chromosome segregation	BP	8.47E−16	6.24E−13	25	271
GO:0000819	Sister chromatid segregation	BP	4.13E−16	4.57E−13	23	213
GO:0000070	Mitotic sister chromatid segregation	BP	2.50E−12	1.11E−09	16	132
GO:0007062	Sister chromatid cohesion	BP	1.28E−11	4.70E−09	15	124
GO:0051321	Meiotic cell cycle	BP	1.09E−06	9.63E−05	13	212
GO:0018205	Peptidyl‐lysine modification	BP	.000711644	.014168764	13	397
GO:0007126	Meiotic nuclear division	BP	4.21E−06	.000282256	11	169
GO:1903046	Meiotic cell cycle process	BP	6.57E−06	.000382253	11	177
GO:0006310	DNA recombination	BP	.000211923	.00578211	11	259
GO:0006302	Double‐strand break repair	BP	.00010557	.003381302	10	199
GO:0034502	Protein localization to chromosome	BP	5.31E−07	5.34E−05	8	62
GO:0007127	Meiosis I	BP	.000129079	.00392795	7	97
GO:0016925	Protein sumoylation	BP	.000772302	.014841624	7	130
GO:0045132	Meiotic chromosome segregation	BP	.001628625	.027267138	5	74
GO:0007131	Reciprocal meiotic recombination	BP	.001756437	.028753532	4	45
GO:0035825	Reciprocal DNA recombination	BP	.001756437	.028753532	4	45
GO:0007064	Mitotic sister chromatid cohesion	BP	.002247081	.035471781	3	23

GO ID: Unique label information in Gene Ontology database; Description: Gene Ontology function description information; Term type is the category of the GO (CC, Cellular component; MF, Molecular function; BP, Biological pathway); *P*‐value < .05, this function is enrichment item; *P*‐adjust: the corrected *P*‐value; DEG item: The number of genes associated with the Term in differentially expressed genes. BG item: The number of genes associated with the Term in all genes.

### 3.8 The influence of knocked‐down and up‐regulated Rad21 gene on XPO1, P53, and cell cycle‐related proteins

**Figure 6:**
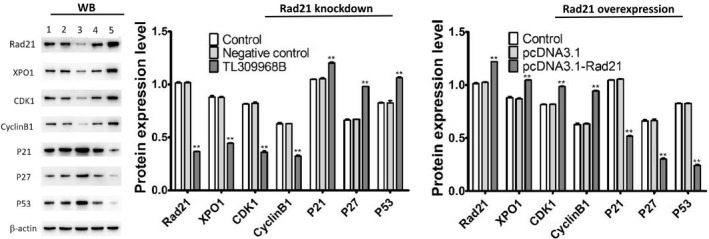
The influence of knocked‐down and up‐regulated Rad21 gene on XPO1, P53 and cell cycle‐related proteins in HeLa cells. Western blot (WB) showed the expression of XPO1, CyclinB1, CDK1, P21, P27, and P53 following Rad21 knockdown and overexpression in HeLa cells. Line 1, 2, 3, 4, 5 represent control, negative control, TL309968B, pcDNA3.1, and pcDNA3.1‐Rad21, respectively. **P < .01 vs the control group

A eukaryotic expression plasmid with Rad21 expression was transfected into HeLa cells to artificially induce Rad21 high expression. When Rad21‐specific shRNA (TL309968B) knocked down Rad21 expression, the expression of XPO1, CyclinB1 and cyclin‐dependent kinase CDK1 were downregulated, in companion with the upregulation of cytokine‐induced protein/kinase interacting protein P21 and P27 and the tumor suppressor gene P53. When artificial high expression of Rad21 through the plasmid, above gene expression was reversed (Figure 6).

## 4 DISCUSSION

### 4.1 Oncogene characteristics of the Rad21 gene and potential targets for radiation treatment in CC

Rad21 is known to be highly expressed in some malignant tumors and is related to the biological behaviors of tumors. However, the role of Rad21 in CC has not been reported. In this study, we found that Rad21 expression increased with the degree of cervical lesions, which is consistent with the results of the Gene Expression Omnibus (GEO) database analysis.^10^ Our results also showed that Rad21 knockdown with its shRNA lentivirus in HeLa and Siha cells results in decreased cell proliferation, colony formation, invasion, and migration, as well as increased apoptosis, cell cycle arrest at the G2/M phase and sensitivity to radiation. The survival data of the TCGA database also show that the survival time associated with high Rad21 expression was significantly shorter than that for low Rad21 expression. Therefore, our results demonstrate that aberrant expression of Rad21 is present in CC and affects the biological behavior of CC through oncogenic activity.

The oncogenic properties of Rad21 have been confirmed in other studies. Atienza et al^9^ showed that the expression of Rad21 in breast cancer cells is 1.25‐2.5 times higher than in normal breast tissue and that knockdown of Rad21 decreases Rad21 mRNA levels, inhibits cell proliferation, promotes apoptosis, and increases the sensitivity of MCF‐7 cells to etoposide and bleomycin. Mahmood et al^8^ also confirmed that Rad21 is one of the driver genes that regulate the proliferation/survival of clonogenic breast cancer cells presenting an amplification of the corresponding region. Yamamoto et al^4^ found that Rad21 is closely related to invasion and metastasis of squamous cell carcinoma of the oral cavity. Recently, Cai et al^11^ found that nuclear Rad21 was increased in hepatocellular carcinoma tissues compared to adjacent nontumor tissues, and higher Rad21 levels are associated with shorter overall survive of hepatocellular carcinoma patients. Increased Rad21 gene dosage is associated with Rad21 mRNA expression and unfavorable tumor characteristics.^12^

The relationship between the Rad21 gene and radiation sensitivity is another important question. Rad21 is a novel mammalian radiation‐responsive gene, which was first reported to be involved in radiation‐induced DNA double‐strand break repair of *Schizosaccharomyces pombe* in 1992.^13^ Xu et al^14^ found heterozygous Rad21+/− mouse embryonic stem cells exhibit homologous recombination (HR) deficiency, indicating that mouse Rad21 is also required for HR. Compared to their control littermates, heterozygous Rad21+/− animals exhibited greater sensitivity to whole body irradiation. Small intestine crypt cells with rapid cell proliferation such as malignant tumors in Rad21+/− mutant animals are more susceptible to killing by radiation. The above study provides a theoretical explanation for targeted depletion of Rad21 function in DNA‐damaged repair to improve the radiosensitivity of HeLa and Siha cells in our study.

### 4.2 The association of polymorphism of Rad21 with survival and susceptibility of CC

Rad21 is an essential gene in mammals, whose loss leads to embryonic death.^14^ Mutations or changes in polymorphisms of Rad21 can cause genomic instability that raises the tumorigenesis possibility. Rad21 mutations and haploin sufficiency reduce Rad21 protein levels and induce severe radiation side effects in normal tissues.^14^, ^15^, ^16^ In 4 tissue samples with Rad21 mutations from TCGA data, Rad21 expression levels in 3 samples are ranked in the lowest 30 cases. These 3 patients were still alive at the time of data collection. TCGA data showed that Rad21 mutations most likely reduce its expression and promote longer survival in CC patients.

Our study showed that women with the rs2289937 C genotype are 2.115 times more likely to develop CC than those with the normal genotype. Alleles of loci rs4570 and rs4579555 were significantly associated with the risk of CC. Haplotypes H1 also increased the risk of CC. Sehl et al^17^ reported that rs16888927, rs16888997, and rs16889040 in introns of Rad21 were associated with breast and ovarian cancer in 104 SNPs of 17 genes associated with double‐strand break repair. Rad21 rs1374297C>G is associated with worse disease‐free survival of early‐stage nonsmall cell lung cancer.^18^ Based on the above study, it is necessary to detect Rad21 polymorphisms and mutations in CC in order to understand susceptibility, disease prognosis and radiotherapeutic sensitivity.

### 4.3 Aberrant high expression of Rad21 is significantly associated with XPO1 expression

Rad21, an important subunit of cohesin, plays an important role in maintaining the correct separation of sister chromatids. It also acts as a target gene for multiple genes or as a regulatory gene for other genes to participate extensively in the physiological and pathological processes of the human body.^19^, ^20^, ^21^ GO analysis (Table 2) for Rad21 coexpressed genes in TCGA data showed that Rad21 as a cellular component relates to chromosomal construction. It possesses a molecular function and involves DNA‐dependent ATPase activity and microtubule motor activity, tubulin and microtubule binding. Rad21 also broadly participates in cellular biological pathways, such as DNA recombination, double‐strand break repair, meiotic and mitotic cell cycle processes, et cetera. More interestingly, the protein interaction network between Rad21 and its coexpressed genes (Figure 3) and KEGG pathway analysis (Figure 4) both reveal that there is a close functional relationship between Rad21 and RNA nuclear transport via XPO1.

The cellular nuclear transport process is critical for gene expression, signal transduction, and oncogenesis. The vast majority of tumor suppressor proteins should be located in the nucleus to exercise their appropriate functions; however, in malignant cells, tumor suppressors tend to be translocated to the cytoplasm, resulting in the inactivation of suppressed proteins and the development of malignant tumors.^22^ Four important factors are related to nuclear transport: nucleoporins, RanGTPase, karyopherins (importin/exportin/transportin), and nuclear localization signals (NLSs) or nuclear export signals (NESs) in cargo molecules. XPO1 is the most important of 8 exportins (exportin‐1‐exportin‐7, exportin‐t) and is involved in the export of cargo molecules.^23^ It has been confirmed that XPO1 exports proteins with leucine‐rich nuclear output signals into the cytoplasm via nuclear pore complexes. It has also been demonstrated that XPO1 participates in exporting various tumor suppressor proteins including retinoblastoma protein, adenomatous polyposis coli, p53, p21, p27, FOXO, IκB, topoisomerase II, and PAR‐4.^24^, ^25^

XPO1 up‐regulation is associated with various cancers including those of female genital organs.^26^ A small molecule inhibitor for exportin‐1 is being tested in a clinical trial.^26^, ^27^ High expression of XPO1 is an independent factor for predicting poor prognosis in ovarian cancer.^28^ XPO1 siRNA‐induced apoptosis of CC cells was accompanied by an increase in the levels of the growth inhibitory proteins p53, p27, p21 and p18.^25^, ^29^ In addition to nuclear export of proteins, XPO1 has been shown to export various RNAs.^30^, ^31^, ^32^, ^33^ In our study, KEGG pathway analysis showed that XPO1 is involved in U snRNA and pre‐rRNA nuclear export. We also demonstrated that the Rad21 influences the expression of XPO1, CyclinB1, CDK1, P21, P27 and P53 through up‐ and downregulating the Rad21 expression, hence suggesting that the Rad21 gene promotes the development of cervical cancer possibly by participating in the regulation of cell cycle and the nuclear output of the tumor suppressor gene by XPO1.

In summary, our study showed that aberrant expression of Rad21 was present in CC and affected the biological behavior of CC through oncogenic activity. Mutations and polymorphisms of Rad21 were associated with survival and susceptibility of CC. There is a close functional relationship between Rad21 and RNA nuclear transport via XPO1. The Rad21 gene involves the development of cervical cancer possibly by participating in the regulation of cell cycle and the nuclear output of the tumor suppressor gene by XPO1. A comprehensive understanding of the molecular interactive mechanism between Rad21 and XPO1 in nuclear transport will lead to better miRNA therapeutic strategies for cervical cancer.

## CONFLICT OF INTERESTS

None declared.
