# Supplementary figures and images for: The effect of aberrant expression and genetic polymorphisms of Rad21 on cervical cancer biology
Source: Cancer Med. 2018 May 24;7(7):3393–405. doi: 10.1002/cam4.1592 (PMC6051231; doi:10.1002/cam4.1592)

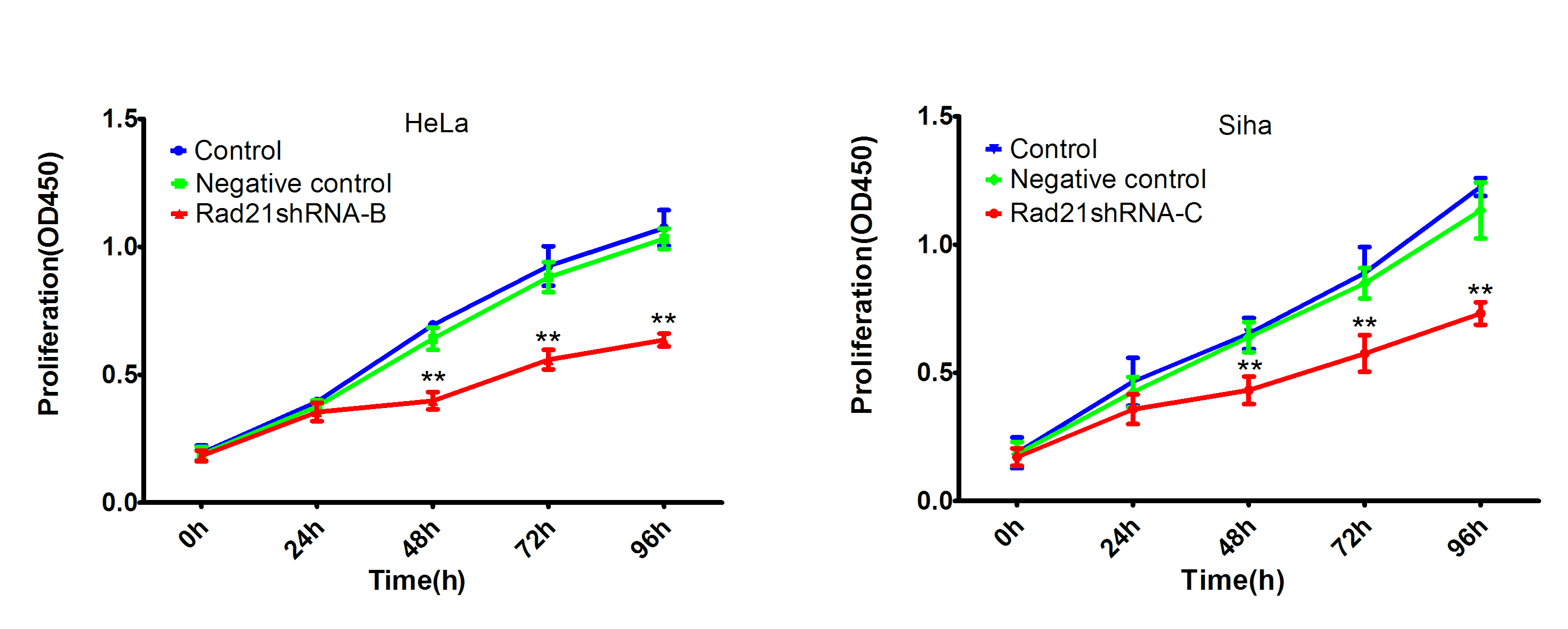

Supplement: Supplementary file 1 [file CAM4-7-3393-s001.tif]
