# Supplementary material for: The effect of aberrant expression and genetic polymorphisms of Rad21 on cervical cancer biology
Source: Cancer Med. 2018 May 24;7(7):3393–405. doi: 10.1002/cam4.1592 (PMC6051231; doi:10.1002/cam4.1592)
Supplement: Supplementary file 6 [file CAM4-7-3393-s006.docx]

Supporting Information, Fig.1. Proliferation curves of HeLa and Siha cells infected with Rad21 shRNA lentivirus. ***P* <0.01 versus the negative control.

Supporting Information, Fig.2. Effect of Rad21 shRNA on cell apoptosis and cycle of HeLa and Siha cells. Flow cytometry was performed to determine cell apoptosis and cycle phase. After 48 hours of culture, apoptosis rates of HeLa cells with stable expression of shRNA TL309968B and Siha cells with stable expression of shRNA TL309968C were higher than in control groups. Compared with scrambled negative control, the ratio of G2/M phase cells increased significantly. ***P* <0.01 versus the scrambled negative control.

Supporting Information, Fig.3. Protein-protein interaction map of Rad21 coexpressed genes. Rad21 interacts with XPO1 via UBC, CDC20, and RANBP2. Cohesin subunit Smc1, Smc3, and STAG interact with XPO1 proteins, in which direct interaction exists between Smc3 and XPO1.

Supporting Information, Fig.4. KEGG analysis of Rad21 coexpressed genes. Rad21 plays a major role in the M phase of the cell cycle and RNA transport in human malignant tumors. As seen from the bottom diagram, an important CRM1 gene in RNA translocation is involved in the RNA transport under the action of RNA polymerase. The top two diagrams are quoted from the KEGG database (bread cancer).
